# Radiographic differences in the concomitant deformities in two types of medial ankle osteoarthritis

**DOI:** 10.1371/journal.pone.0247816

**Published:** 2021-03-03

**Authors:** Wooyoung Choi, Chin Youb Chung, Moon Seok Park, Sanghoon Lee, Kyoung Min Lee

**Affiliations:** Department of Orthopedic Surgery, Seoul National University Bundang Hospital, Seongnam, South Korea; MD Anderson Cancer Center, UNITED STATES

## Abstract

**Objectives:**

Motion preserving surgeries could be unsuccessful because of underestimation of deformities of the foot and knee in ankle osteoarthritis. This study aimed to investigate the concomitant deformities in medial ankle osteoarthritis and the difference between the two types, varus angulation and medial translation.

**Methods:**

A retrospective study was conducted using medical records and radiographic data. Patients with medial ankle osteoarthritis that underwent weight-bearing X ray imaging and radiographic measurements including tibial plafond inclination (TPI), tibiotalar tilt angle (TT), lateral talo-first metatarsal angle, naviculo-cuboid overlap, and mechanical tibiofemoral angle (mTFA) were studied. The patients were categorized into two groups, the varus angulation group (TT ≥4°) and medial translation group (TT <4°). The radiographic measurements were compared between the two groups.

**Results:**

A total of 102 patients (male = 44; female = 58) were included; the mean age was 64.9 years (SD 8.3 years). The varus rotation group (N = 66) showed a significantly smaller lateral talo-first metatarsal angle (p<0.001), naviculo-cuboid overlap (p<0.001), and mTFA (p = 0.019) compared to the medial displacement group (N = 36). The TT showed a significant correlation with lateral talo-first metatarsal angle (r = -0.520, p<0.001), naviculo-cuboid overlap (r = -0.501, p<0.001), and mTFA (r = -0.243, p = 0.014). Lateral talo-first metatarsal angle was found to be the significant factor (p = 0.018) discriminating varus angulation and medial translation types in the binary logistic analysis.

**Conclusions:**

Varus angulation of the ankle was correlated with knee alignment and foot deformity. Radiographic indices were different between the varus angulation and medial translation groups. The role of concomitant deformities needs to be further investigated in terms of a causal relationship. Surgeons need to pay attention to concomitant deformities in the treatment of medial ankle osteoarthritis.

## Introduction

Ankle osteoarthritis is a degenerative disease causing pain and limitation of daily activities, which eventually impedes a patient’s quality of life [1]. The prevalence of symptomatic ankle osteoarthritis has been reported to be 3.4% [2], and it is expected to increase with an aging population. With the recent developments in surgical techniques and an in-depth understanding of ankle biomechanics, the surgical outcomes of ankle osteoarthritis have improved [3]. However, the surgical outcome of a total ankle replacement or a supramalleolar osteotomy is occasionally unfavorable [4].

As a result of movement through ambulation or sports activity that impacts the ankles, ground reaction force is transmitted to the ankle and other parts of the body through the foot. Upper body weight is also transmitted to the ankle joint through the mechanical axis [5]. Therefore, foot shape, deformities and disturbances in the mechanical alignment of the lower extremity can directly affect the pattern of force transmission to the ankle joint causing abnormal stress on specific parts of the joint. This in turn can affect cartilage wear and tear patterns or pose as a predisposing factor for ankle injuries [6]. It is well known that a deformity in the heel varus or a cavus foot can cause ankle inversion injuries [7], however, the evidence showing a causative relationship between foot deformities and specific patterns of ankle cartilage wear and tear in ankle osteoarthritis is limited.

Concomitant deformities of the foot or knee joint in medial ankle osteoarthritis have not been sufficiently documented. Furthermore, there is no clear indication to correct a foot deformity when performing motion preserving surgeries such as a total ankle arthroplasty or a supramalleolar osteotomy for ankle osteoarthritis. The authors consider that an insufficient understanding of the deformities associated with ankle osteoarthritis could be the cause of an unfavorable surgical outcome of a total ankle arthroplasty or a supramalleolar osteotomy. As such, inappropriately or insufficiently addressed deforming forces from the adjacent parts of the body could still increase contact pressure in the joint, leading to accelerated implant wear or progression of osteoarthritis [8].

Therefore, the purpose of this study was to investigate concomitant foot deformities and the mechanical axis of the lower extremity in patients with medial ankle osteoarthritis in a retrospective cohort at a single institution.

## Materials and methods

This study was approved by the institutional review board at Seoul National University Bundang Hospital (IRB No. B-2004-606-107) and the requirement of informed consent from the participants was waived due to the retrospective nature of the study.

### Subjects

We reviewed and retrieved the information of consecutive patients with ankle osteoarthritis who visited our foot and ankle clinic between January 2017 and December 2019. The patients with ankle osteoarthritis underwent weight-bearing ankle anteroposterior (AP), lateral foot and ankle and mortis view X rays as well as standing full-limb AP radiographs. Of these, patients who were diagnosed with medial ankle osteoarthritis on radiographic examination were selected. Medial ankle osteoarthritis was defined as joint space narrowing on the medial gutter or that between the medial talar dome and the medial tibial plafond. The exclusion criteria were as follow: 1) congenital anomaly, 2) neuromuscular diseases, 3) tumor, 4) infection, 5) avascular necrosis, 6) rheumatoid arthritis or inflammatory arthritis, 7) previous fracture, 8) previous foot, ankle or knee surgeries, and 9) any other conditions that could change the normal anatomy of the lower extremity other than osteoarthritis. The demographic data of the patients collected included age, sex, body mass index (BMI) etc.

### Radiographic examination

The radiographs of the patients were captured using a UT 2000 X-ray machine (Philips Research, Eindhoven, the Netherlands) according to our protocol which is as follows: The weight-bearing AP view of the ankles was obtained with the horizontal beam centered between the ankle joints at joint level. The patient was positioned on a 5-cm block, with the film cassette behind the heels. The weight-bearing lateral view of the foot and ankle was captured separately for each foot in the standing position with the beam focusing on the lateral malleolus. The patient was placed in the erect position and the cassette was positioned between both feet. The mortise view was obtained by internally rotating the foot 15 to 20 degrees to bring the talus into its true AP position and the malleoli equidistant from the cassette. The primary beam was centered on the joint space, and the foot was dorsiflexed to avoid the tip of the lateral malleolus being overlapped by the calcaneus. The radiograph setting was 60 kVp and 10 mAs at a source-to-image distance of 110 cm. The teleradiogram was obtained by vertically entering the horizontal center beam to the patella height and vertical beam to the midline. Full-length standing AP radiographs were obtained on a 14×51-inch grid cassette at a source to image distance of 240 cm with a setting of 90 kV and 50 mAs. All radiographic images were digitally acquired using a picture archiving and communication system (PACS; Infinitt, Seoul, South Korea), and radiographic measurements were performed using PACS software.

### Radiographic measurements and interobserver reliability

Four radiographic measurements evaluating the extent of ankle arthritis and the foot deformity were analyzed based on evidence highlighted after the literature review had been conducted: tibial plafond inclination (TPI) [9], tibiotalar tilt (TT) [10–13], lateral talo-first metatarsal angle [13–15], naviculo-cuboid overlap [14, 16, 17], and mechanical tibiofemoral angle (mTFA) [18].

**Fig 1:**
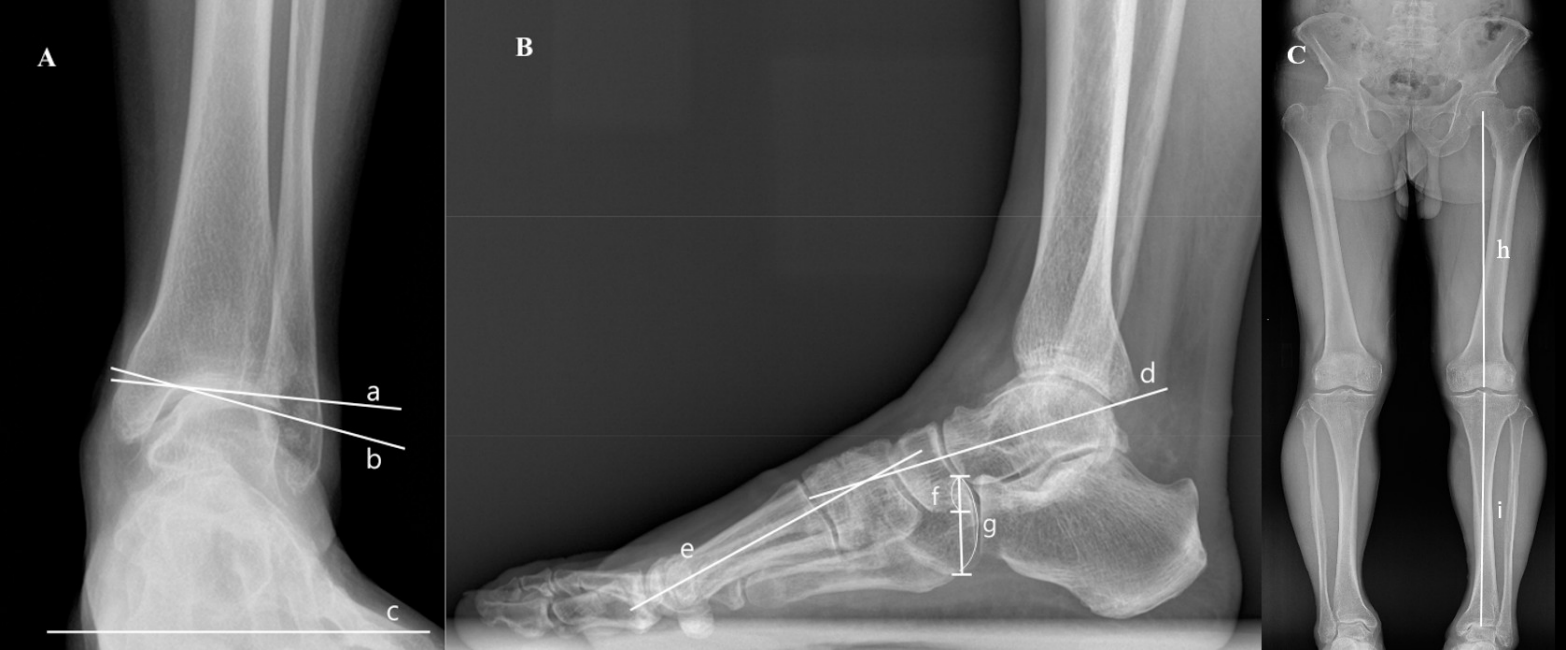
Radiographic measurements. A: The ankle in AP view, TPI is the angle between the tibial plafond (a) and the floor (c). The TT is measured between the tibial plafond (a) and the talar dome (b). B: The foot and ankle in lateral view, the lateral talo-first metatarsal angle is the angle between the longitudinal axis of the talus (d) and the first metatarsal (e). The naviculo-cuboid overlap is the overlapped portion between the navicular and cuboid (f) divided by the vertical height of the cuboid (g). C: The mechanical tibiofemoral angle is the angle between the mechanical axis of the femur (h) and that of the tibia (i).

On the AP view, the TPI was the angle between the tibial plafond and the horizontal line. The TT was the angle between the tibial plafond and the talar dome (Fig 1A). On the lateral view, the lateral talo-first metatarsal angle was the angle between the longitudinal axes of the talus and the first metatarsal. The naviculo-cuboid overlap was the overlapped portion of the navicular and the cuboid divided by the vertical height of the cuboid (Fig 1B). The mechanical tibiofemoral angle (mTFA) was the angle between the mechanical axis of the femur (a line connecting the center of the femoral head and the center of the intercondylar notch) and that of the tibia (a line connecting the center of the tibial spines and the center of the tibial plafond) (Fig 1C).

Two orthopedic surgeons, with 5 and 2 years’ worth of orthopedic experience, participated in the interobserver reliability testing of the radiographic measurements after a consensus building session. Each surgeon performed radiographic measurements for a predetermined number of radiographic images that were presented in random order by a research assistant who was not involved in this study. Following reliability testing, one of the two surgeons (with 5 years of experience) measured the radiographic parameters for all patients.

### Classification of ankle arthritis

**Fig 2:**
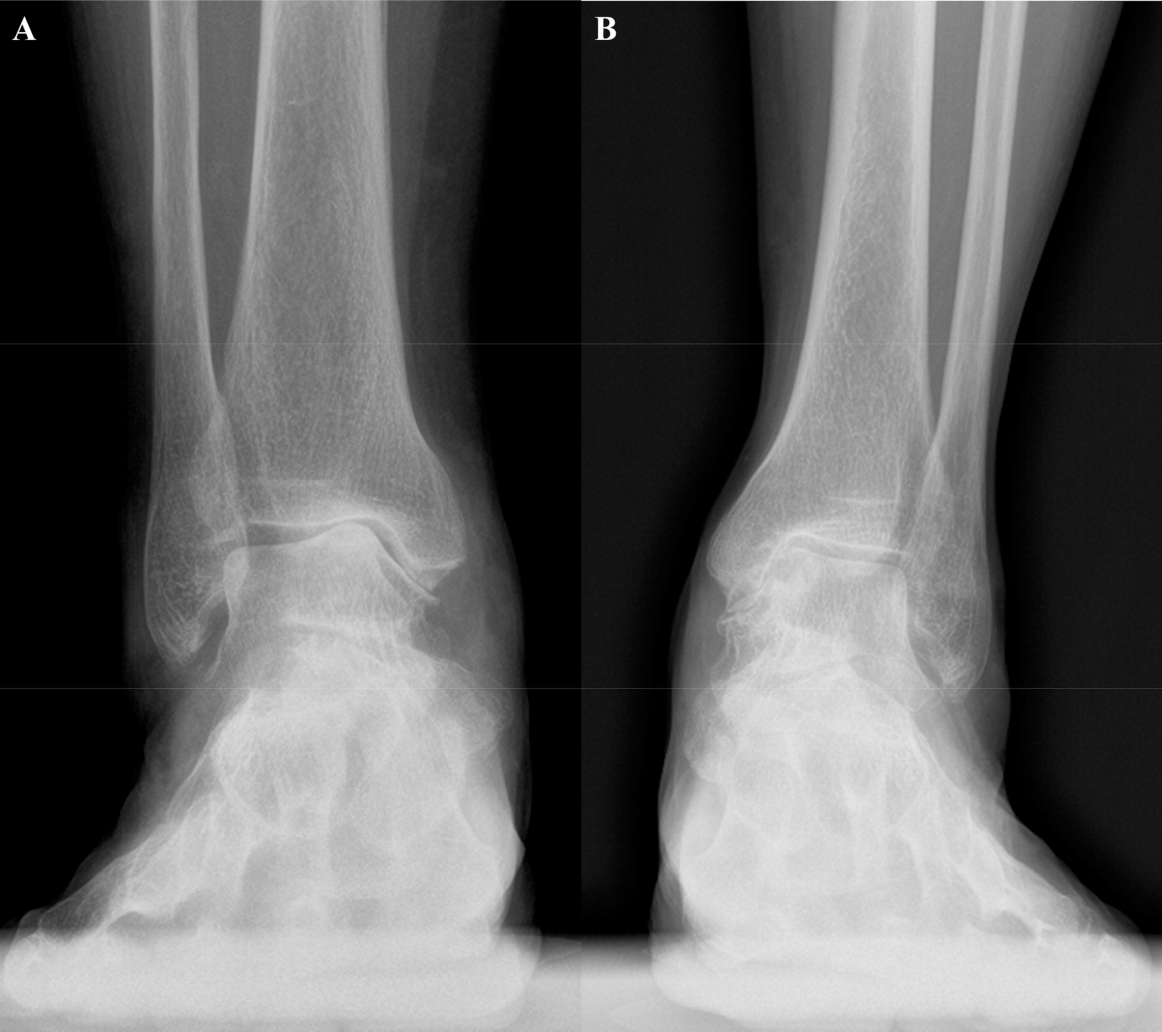
Two types of medial ankle osteoarthritis. A: The varus angulation type is defined as a TT ≥ 4°. B: The medial translation type as TT < 4°.

Medial ankle osteoarthritis was divided into the varus angulation type and the medial translation type according to the primary area of joint space narrowing. The medial gutter was primarily narrowed in the medial translation type and upper joint space between the medial talar dome and the medial tibial plafond was primarily narrowed in the varus angulation type (Fig 2). In cases where the classification was ambiguous because the joint space was narrowed in both, the medial gutter and the upper joint space, the patients with a TT ≥ 4° were classified as the varus angulation type and those with a TT < 4° as the medial translation type [3, 19].

### Statistical analysis

A descriptive statistical analysis was performed including the average, standard deviation (SD), and proportion. Data normality was determined by the Kolmogorov-Smirnov test. Comparison of means between the varus angulation type and the medial translation type was performed using a Student t-test. Proportion analysis between the two groups was conducted by the chi-square test. The correlation between the variables was analyzed using the Pearson’s correlation coefficient. Binary logistic analysis was performed to identify the significant radiographic factors (other than the TT angle) that determined the varus angulation type and the medial translation type in patients with medial ankle osteoarthritis.

Interobserver reliability was tested using the intraclass correlation coefficient (ICC) with a two-way random effect model, assuming a single measurement and absolute agreement. The sample size for reliability test was calculated with an ICC target value of 0.8 and 95% confidence interval (CI) width of 0.2. A minimum number of interobserver reliability for the two raters was 52 as was determined by Bonnett’s approximation [20]. All statistical analyses were performed using SPSS version 20.0 (IBM Corp., Armonk, NY, USA), and statistical significance was accepted when the p-values were <0.05.

## Results

A total of 102 patients with medial ankle osteoarthritis were included in the analysis. The mean age of the patients was 64.9 years (SD 8.3 years), and there were 44 men and 58 women. The mean BMI was 26.4 kg/m^2^ (SD 2.7 kg/m^2^) for men and 27.5 kg/m^2^ (SD 3.0 kg/m^2^) for women. There were 55 right and 47 left arthritic ankles.

Interobserver reliabilties of TPI, TT, lateral talo-first metatarsal angle, naviculo-cuboid overlap and mTFA were 0.886 (95% CI, 0.800 to 0.935), 0.850 (95% CI, 0.750 to 0.912), 0.891 (95% CI, 0.794 to 0.941), 0.885 (95% CI, 0.804 to 0.933), and 0.958 (95% CI, 0.927 to 0.976), respectively.

The TT angle (p<0.001), the lateral talo-first metatarsal angle (p<0.001), naviculo-cuboid overlap (p<0.001), and mTFA (p = 0.019) were significantly different between the varus angulation and the medial translation groups in medial ankle osteoarthritis (Table 1).

**Table 1:** Comparison of variables between the varus angulation and the medial translation groups.

	Varus angulation	Medial translation	P value
No. of patients	66	36	-
Age (years)	65.3 (SD 8.6)	64.2 (SD 7.8)	0.528
Men : Women	33 : 33	11 : 25	0.864
Right : Left	36 : 30	19 : 17	0.058
BMI (kg/m^2^)	27.2 (SD 3.3)	26.5 (SD 1.9)	0.391
Radiographic measures			
TPI (°)	6.6 (SD 3.8)	7.3 (SD 2.9)	0.302
TT (°)	9.1 (SD 4.9)	1.5 (SD 1.1)	<0.001
Lateral talo-1MT (°)	-8.5 (SD 12.4)	4.2 (SD 8.0)	<0.001
NC overlap	0.36 (SD 0.17)	0.53 (SD 0.14)	<0.001
mTFA (°)	1.8 (SD 4.0)	3.8 (SD 3.7)	0.019

No., number; SD, standard deviation; BMI, body mass index; TPI, tibial plafond inclination; TT, tibiotalar tilt angle; Lateral talo-1MT, lateral talo-first metatarsal angle; NC overlap, naviculo-cuboid overlap; mTFA, mechanical tibiofemoral angle.

The TT angle showed a significant correlation with the lateral talo-first metatarsal angle (r = -0.520, p<0.001), the naviculo-cuboid overlap (p = -0.501, p<0.001), and the mTFA (r = -0.243, p = 0.014). The lateral talo-first metatarsal angle showed a significant correlation with the naviculo-cuboid overlap (r = 0.752, p<0.001). The mTFA showed a significant correlation with the TPI (r = 0.369, p<0.001), the lateral talo-first metatarsal angle (r = 0.424, p<0.001), and naviculo-cuboid overlap (r = 0.328, p = 0.001) (Table 2).

**Table 2:** Correlation coefficients between the radiographic measurements.

	TPI	TT	Lateral talo-1MT	NC overlap
TT	-0.194 (p = 0.051)			
Lateral talo-1MT	0.172 (p = 0.083)	-0.520 (p<0.001)		
NC overlap	0.193 (p = 0.052)	-0.501 (p<0.001)	0.752 (p<0.001)	
mTFA	0.369 (p<0.001)	-0.243 (p = 0.014)	0.424 (p<0.001)	0.328 (p = 0.001)

TPI, tibial plafond inclination; TT, tibiotalar tilt angle; Lateral talo-1MT, lateral talo-first metatarsal angle; NC overlap, naviculo-cuboid overlap; mTFA, mechanical tibiofemoral angle.

The lateral talo-first metatarsal angle, the naviculo-cuboid overlap and mTFA were the candidate radiographic factors that determined whether the medial ankle osteoarthritis classified as the varus angulation type or the medial translation type in the binary logistic regression analysis, and lateral talo-first metatarsal angle was found to be the significant factor (p = 0.018) when adjusted for other radiographic measurements (Table 3).

**Table 3:** Significant factors discriminating between the varus angulation and medial translation groups in medial ankle osteoarthritis.

	B	SE	Wald	Exp (B)	P value
TPI	0.007	0.072	0.009	1.007	0.925
Lateral talo-1MT	0.081	0.034	5.619	1.085	0.018
NC overlap	2.960	2.061	2.063	19.301	0.151
mTFA	0.015	0.073	0.045	1.016	0.832
constant	-1.871	1.109	2.848	0.154	0.091

SE, standard error; TPI, tibial plafond inclination; Lateral talo-1MT, lateral talo-first metatarsal angle; NC overlap, naviculo-cuboid overlap; mTFA, mechanical tibiofemoral angle.

## Discussion

This study investigated concomitant deformities of the foot and knee in patients with medial ankle osteoarthritis and the differences between the varus angulation and medial translation types. Deformity at the ankle joint (TT) was found to be correlated with knee deformity (mTFA) and foot deformity (lateral talo-first metatarsal angle and naviculo-cuboid overlap). The alignment of the knee and foot were significantly different between the varus angulation and medial translation types.

In the 102 patients included in this study with medial ankle osteoarthritis, mTFA showed a significant correlation with TT angle, the lateral talo-first metatarsal angle, and naviculo-cuboid overlap. This represented that a greater genu varum deformity correlated with a lesser varus incongruency of the ankle joint, a greater pes planovalgus deformity, and greater midfoot pronation. This implies that deformities above and below the ankle joint may compensate for each other to maintain the plantigrade foot position during weight-bearing motion [13], and this might exert abnormal biomechanical forces on the ankle joint between the foot and knee.

**Fig 3:**
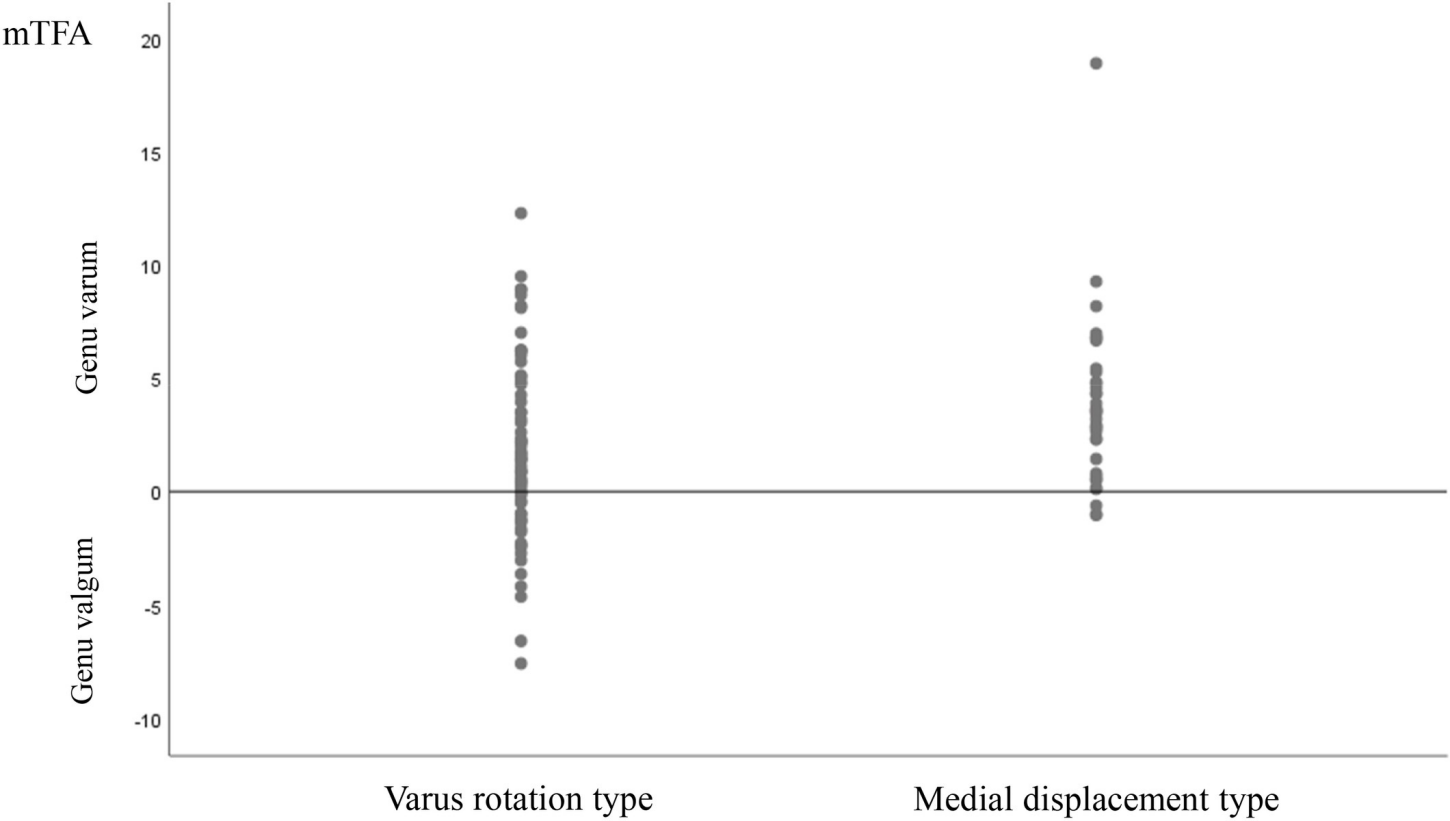
The mTFA of the varus angulation type tends to be distributed in the genu valgum to a greater extent compared to that of the medial translation type.

Although the mTFA showed a mean difference of only 2° between the varus angulation and medial translation types, the distribution of mTFA was quite different. In the varus angulation group it tended to be distributed in the genu valgum compared to the medial translation group (Fig 3). As of now, a causal relationship between the genu valgum and the varus ankle incongruency is not clear, and the pathogenetic effect of the genu valgum deformity on the varus incongruency of the ankle joint should be further investigated in a cadaver or biomechanical study.

A previous study have reported unfavorable surgical outcomes following a supramalleolar osteotomy in patients with a larger TT angle [21]. The authors reckon that varus angulation and medial translation types might exhibit a different pathophysiology in the development of medial ankle osteoarthritis. Binary logistic regression analysis showed that lateral talo-first metatarsal angle was the only significant factor discriminating the varus angulation and medial translation groups. Therefore, when the weight-bearing lateral ankle X rays were performed, the foot should be included and the lateral talo-first metatarsal angle measurement should be evaluated preoperatively in patients with medial ankle osteoarthritis.

The varus angulation type showed a significantly smaller lateral talo-first metatarsal angle and naviculo-cuboid overlap than the medial translation type, which represents a high medial foot arch and midfoot supination. These deformities are usually concurrent with hindfoot varus deformity. It is unknown as to whether these are reversible and compensatory deformities in response to talar varus or a true rigid deformity. Considering that an initial flexible deformity can develop into a rigid deformity over time [22], the high medial foot arch and heel varus deformity need to be further examined preoperatively and this would include the Coleman block test [23] in patients with medial ankle osteoarthritis, especially for the varus angulation type.

**Fig 4:**
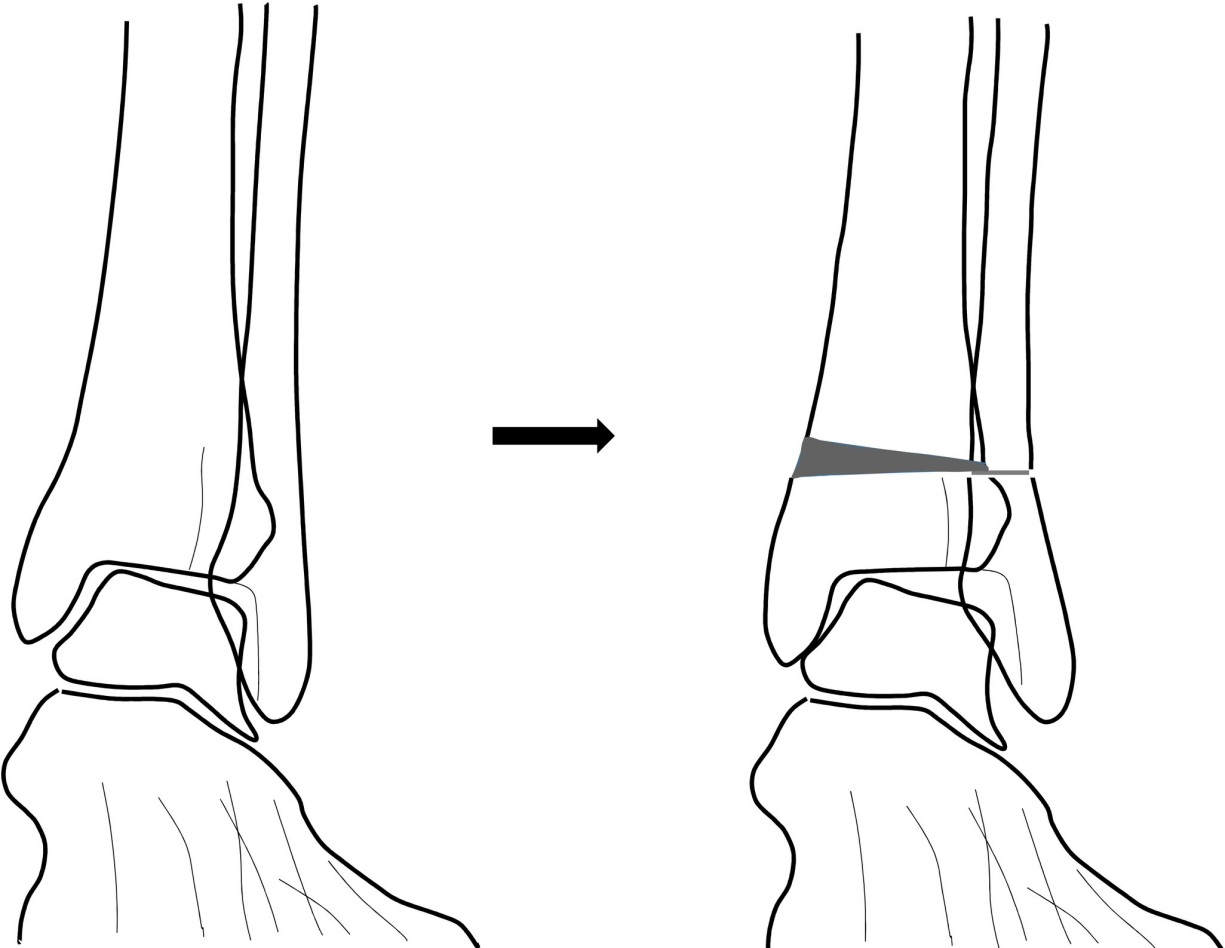
In patients with rigid foot varus deformities, corrective osteotomy of the distal tibia to a more valgus alignment can paradoxically aggravate ankle varus incongruency or TT because the foot cannot compensate and adapt to changes in mechanical alignment.

Unlike the flexible varus deformity of the foot (heel varus, midfoot supination, and a high medial foot arch), a rigid foot varus is unable to compensate for the change in mechanical alignment at or above the ankle joint. Therefore, a supramalleolar valgus osteotomy can possibly aggravate the ankle varus incongruency without correcting the rigid foot varus deformity (Fig 4). Although a previous study have reported a clinically satisfactory surgical outcome following a supramalleolar osteotomy, most of them have failed to correct a large TT angle [21]. However, two studies reported a prominent correction of the TT angle, which was achieved by performing additional procedures that corrected the foot deformity including a calcaneal lateral displacement osteotomy, closing the 1^st^ dorsal metatarsal or a medial cuneiform osteotomy, and other soft tissue procedures [12, 24]. This reflects the importance of correcting deformities of the foot in order to achieve positive outcomes from a supramalleolar osteotomy in patients with medial ankle osteoarthritis especially in the varus angulation type. Therefore, the importance of evaluating and treating deformities of the foot should be highlighted in the surgical treatment of medial ankle osteoarthritis.

There are some limitations to our study that should be addressed. First, this was a retrospective study in a single institution, and unknown bias might have affected the study results. Second, although the deformity of the foot, ankle, and knee is three-dimensional, this study did not include an evaluation of the deformity in the transverse plane. The AP view of the foot and heel alignment should be included, and ideally, a weight-bearing three-dimensional evaluation of the whole extremity would provide a more comprehensive insight into various deformities of the foot, ankle, and knee. As such, these measurements should also be included in future studies. However, a previous study showed that the lateral talo-first metatarsal angle and the naviculo-cuboid overlap were reliable and valid parameters to evaluate hindfoot varus and valgus deformities [25]. Third, this study focused on radiographic data and did not include any clinical information such as patients’ pain, medication usage and quality of life. Fourth, although this study demonstrated the radiographic differences between the two types of medial ankle osteoarthritis, the different pathogeneses could not be accurately explained. Further biomechanical studies are required to support our study results.

In conclusion, medial ankle osteoarthritis was found to be concomitant with foot or knee deformities. Further studies are required to elucidate whether the deformities are flexible and compensatory following the development of medial ankle osteoarthritis or if they are true rigid deformities that cause medial ankle osteoarthritis. The varus angulation type and medial translation type might have a different pathophysiology and therefore may require different surgical strategies.

## References

[1] Valderrabano V, Nigg BM, von Tscharner V, Stefanyshyn DJ, Goepfert B, Hintermann B. Gait analysis in ankle osteoarthritis and total ankle replacement. Clin Biomech (Bristol, Avon). 2007 10;22(8):894–904. 10.1016/j.clinbiomech.2007.05.003 17604886

[2] Murray C, Marshall M, Rathod T, Bowen CJ, Menz HB, Roddy E. Population prevalence and distribution of ankle pain and symptomatic radiographic ankle osteoarthritis in community dwelling older adults: A systematic review and cross-sectional study. PLoS One. 2018 4;13(4):e0193662. 10.1371/journal.pone.0193662 29708977 PMC5927448

[3] Barg A, Pagenstert GI, Horisberger M, Paul J, Gloyer M, Henninger HB, et al. Supramalleolar osteotomies for degenerative joint disease of the ankle joint: indication, technique and results. Int Orthop. 2013 9;37(9):1683–95. 10.1007/s00264-013-2030-2 23959222 PMC3764298

[4] Bloch B, Srinivasan S, Mangwani J. Current Concepts in the Management of Ankle Osteoarthritis: A Systematic Review. J Foot Ankle Surg. 2015 Sep-Oct;54(5):932–9. 10.1053/j.jfas.2014.12.042 26028603

[5] Kakkar R, Siddique MS. Stresses in the ankle joint and total ankle replacement design. Foot Ankle Surg. 2011 6;17:58–63. 10.1016/j.fas.2011.02.002 21549973

[6] Golditz T, Steib S, Pfeifer K, Uder M, Gelse K, Janka R, et al. Functional ankle instability as a risk factor for osteoarthritis: using T2-mapping to analyze early cartilage degeneration in the ankle joint of young athletes. Osteoarthritis Cartilage. 2014 10;22(10):1377–85. 10.1016/j.joca.2014.04.029 24814687

[7] Morrison KE, Kaminski TW. Foot characteristics in association with inversion ankle injury. J Athl Train. 2007 Jan-Mar;42(1):135–42. 17597955 PMC1896068

[8] Joo SD, Lee KB. Comparison of the outcome of total ankle arthroplasty for osteoarthritis with moderate and severe varus malalignment and that with neutral alignment. Bone Joint J. 2017 10;99-B(10):1335–42. 10.1302/0301-620X.99B10.BJJ-2016-1275.R1 28963155

[9] Xie K, Jiang X, Han X, Ai S, Qu X, Yan M. Association Between Knee Malalignment and Ankle Degeneration in Patients With End-Stage Knee Osteoarthritis. J Arthroplasty. 2018 12;33(12):3694–98. 10.1016/j.arth.2018.08.015 30197215

[10] Pagenstert GI, Hintermann B, Barg A, Leumann A, Valderrabano V. Realignment surgery as alternative treatment of varus and valgus ankle osteoarthritis. Clin Orthop Relat Res. 2007 9;462:156–68. 10.1097/BLO.0b013e318124a462 17563701

[11] Barg A, Saltzman CL. Joint-Preserving Procedures in Patients with Varus Deformity: Role of Supramalleolar Osteotomies. Foot Ankle Clin. 2019 6;24(2):239–64. 10.1016/j.fcl.2019.02.004 31036267

[12] Lee WC, Ahn JY, Cho JH, Park CH. Realignment surgery for severe talar tilt secondary to paralytic cavovarus. Foot Ankle Int. 2013 11;34:1552–59. 10.1177/1071100713497001 23832713

[13] Lee WC, Moon JS, Lee HS, Lee K. Alignment of ankle and hindfoot in early stage ankle osteoarthritis. Foot Ankle Int. 2011 7;32(7):693–9. 10.3113/FAI.2011.0693 21972764

[14] Davids JR, Gibson TW, Pugh LI. Quantitative segmental analysis of weight-bearing radiographs of the foot and ankle for children: normal alignment. J Pediatr Orthop. 2005 Nov-Dec;25(6):769–76. 10.1097/01.bpo.0000173244.74065.e4 16294134

[15] Haraguchi N, Ota K, Tsunoda N, Seike K, Kanetake Y, Tsutaya A. Weight-bearing-line analysis in supramalleolar osteotomy for varus-type osteoarthritis of the ankle. J Bone Joint Surg Am. 2015 2;97(4):333–9. 10.2106/JBJS.M.01327 25695986

[16] Ruiz-Picazo D, Jiménez-Ortega P, Doñate-Pérez F, Gaspar-Aparicio N, García-Martín V, Ramírez-Villaescusa J, et al. Radiographic and Functional Results following Subtalar Arthroereisis in Pediatric Flexible Flatfoot. Adv Orthop. 2019 8;2019:5061934. 10.1155/2019/5061934 31467723 PMC6699253

[17] Okamoto Y, Otsuki S, Jotoku T, Nakajima M, Neo M. Clinical usefulness of hindfoot assessment for total knee arthroplasty: persistent post-operative hindfoot pain and alignment in pre-existing severe knee deformity. Knee Surg Sports Traumatol Arthrosc. 2017 8;25(8):2632–9. 10.1007/s00167-016-4122-1 27056693

[18] Lee KM, Chang CB, Park MS, Kang SB, Kim TK, Chung CY. Changes of knee joint and ankle joint orientations after high tibial osteotomy. Osteoarthritis Cartilage. 2015 2;23(2):232–8. 10.1016/j.joca.2014.11.001 25450843

[19] Seki H, Ogihara N, Kokubo T, Suda Y, Ishii K, Nagura T. Visualization and quantification of the degenerative pattern of the talus in unilateral varus ankle osteoarthritis. Sci Rep. 2019 11;9(1):17438. 10.1038/s41598-019-53746-6 31767944 PMC6877636

[20] Zou GY. Sample size formulas for estimating intraclass correlation coefficients with precision and assurance. Stat Med. 2012 12;31(29):3972–81. 10.1002/sim.5466 22764084

[21] Lee WC. Extraarticular Supramalleolar Osteotomy for Managing Varus Ankle Osteoarthritis, Alternatives for Osteotomy: How and Why? Foot Ankle Clin. 2016 3;21(1):27–35. 10.1016/j.fcl.2015.09.002 26915776

[22] Flores DV, Mejia Gomez C, Fernandez Hernando M, Davis MA, Pathria MN. Adult Acquired Flatfoot Deformity: Anatomy, Biomechanics, Staging, and Imaging Findings. Radiographics. 2019 Sep-Oct;39(5):1437–60. 10.1148/rg.2019190046 31498747

[23] Dreher T, Hagmann S, Wenz W. Reconstruction of multiplanar deformity of the hindfoot and midfoot with internal fixation techniques. Foot Ankle Clin. 2009 9;14(3):489–531. 10.1016/j.fcl.2009.06.001 19712887

[24] Lee HS, Wapner KL, Park SS, Kim JS, Lee DH, Sohn DW. Ligament reconstruction and calcaneal osteotomy for osteoarthritis of the ankle. Foot Ankle Int. 2009 6;30(6):475–80. 10.3113/FAI.2009.0475 19486622

[25] Lee KM, Chung CY, Park MS, Lee SH, Cho JH, Choi IH. Reliability and validity of radiographic measurements in hindfoot varus and valgus. J Bone Joint Surg Am. 2010 10; 92(13):2319–27. 10.2106/JBJS.I.01150 20926727

